# A *de novo* chromosome-scale assembly of the *Lablab purpureus* genome

**DOI:** 10.3389/fpls.2024.1347744

**Published:** 2024-03-05

**Authors:** Wirulda Pootakham, Prakit Somta, Wasitthee Kongkachana, Chaiwat Naktang, Chutima Sonthirod, Sonicha U-Thoomporn, Thippawan Yoocha, Poompat Phadphon, Sithichoke Tangphatsornruang

**Affiliations:** ^1^ National Center for Genetic Engineering and Biotechnology (BIOTEC), National Science and Technology Development Agency (NSTDA), Pathum Thani, Thailand; ^2^ Department of Agronomy, Faculty of Agriculture at Kamphaeng Saen, Kasetsart University, Nakhon Pathom, Thailand

**Keywords:** *Lablab purpureus*, genome assembly, Hi-C, chromosome-scale, annotation

## Abstract

**Introduction:**

Lablab (*Lablab purpureus* (L.) Sweet), an underutilized tropical legume crop, plays a crucial role in global food and nutritional security. To enhance our understanding of its genetic makeup towards developing elite cultivars, we sequenced and assembled a draft genome of *L. purpureus* accession PK2022T020 using a single tube long fragment read (stLFR) technique.

**Results and discussion:**

The preliminary assembly encompassed 367 Mb with a scaffold N50 of 4.3 Mb. To improve the contiguity of our draft genome, we employed a chromatin contact mapping (Hi-C) approach to obtain a pseudochromosome-level assembly containing 366 Mb with an N50 length of 31.1 Mb. A total of 327.4 Mb had successfully been anchored into 11 pseudomolecules, corresponding to the haploid chromosome number in lablab. Our gene prediction recovered 98.4% of the highly conserved orthologs based on the Benchmarking Universal Single-Copy Orthologs (BUSCO) analysis. Comparative analyses utilizing sequence information from single-copy orthologous genes demonstrated that *L. purpureus* diverged from the last common ancestor of the *Phaseolus/Vigna* species approximately 27.7 million years ago. A gene family expansion analysis revealed a significant expansion of genes involved in responses to biotic and abiotic stresses. Our high-quality chromosome-scale reference assembly provides an invaluable genomic resource for lablab genetic improvement and future comparative genomics studies among legume species.

## 1 Introduction

Lablab (also known as hyacinth bean; *Lablab purpureus* (L.) Sweet) is an important tropical legume crop of the world. Cultivated extensively as field and vegetable crops by small-scale farmers across tropical and sub-tropical regions of Africa and Asia, lablab serves various purposes, including forage, cover, and green manure crops (Kongjaimun et al., 2023). Its resilience to diverse soil types and climates, including tolerance to drought, salinity, and high temperatures (Pengelly and Maass, 2001), makes it a crucial crop species for global food security. Mature and young seeds as well as pods are widely consumed, and the young leaves are also edible. With dry seeds containing approximately 25% protein, 60% carbohydrate and several essential amino acids and micronutrients, lablab holds significant nutritional value (Hossain et al., 2016; Kala et al., 2010; Hardallo et al., 1980; Shaahu et al., 2015).

Amidst growing concerns regarding climate change and an expanding global population, there has been a concerted effort in recent years to explore underutilized crops for food security. Due to its high nutrition, multi-purposed uses and drought tolerance, lablab emerges as a promising legume species for ensuring food and nutritional security in tropical and subtropical regions (Kongjaimun et al., 2023). However, despite its long history of domestication, lablab still lacks certain desirable domestication and agronomic traits thereby reducing its overall agronomic value. For instance, in Thailand, all lablab cultivars exhibit bushy, trailing or twining characteristics with indeterminate growth habits and are sensitive to day length (Amkul et al., 2021). Consequently, there is a pressing need for lablab genetic improvement to obtain cultivars that are not only productive and highly nutritious, but also resilient to unpredictable climate change.

Despite being a versatile crop, lablab’s potential in addressing food security challenges remains underexploited. Lablab cultivars grown globally are primarily landraces or pure lines selected from landraces, except in a few countries where improved cultivars have been developed through breeding initiatives (Kongjaimun et al., 2023). Existing breeding programs for lablab, mostly small and local in developing and underdeveloped countries, could benefit tremendously from genomic-assisted breeding. Currently, there has only been one published genetic linkage map for lablab (Konduri et al., 2000) and no quantitative trait locus reported so far. Nevertheless, with advances in DNA sequencing technologies, the lablab genome has been sequenced and assembled (Chang et al., 2019; Njaci et al., 2023). The cultivar sequenced by Chang et al. (2019) was not known whereas the cultivar sequenced by Njaci et al. (2023) was Highworth, an accession originated from South India and widely cultivated in Australia for dry seeds/pulse and forage production (Norman, 1990). As different accessions in the germplasm belonging to the same species are likely to have slightly different genome contents and structures as well as gene numbers, a single high-quality reference genome is likely to be inadequate in representing the full spectrum of genome variations in this species (Editorial, 2020). In this study, we generated a chromosome-scale assembly of lablab accession PK2022T020, a landrace cultivar commonly grown for vegetable pods/seeds consumption in Thailand. We specifically chose this local cultivar due to its agronomically desirable traits including rapid and vigorous growth, late flowering (sensitive to day length), perennial-like habit and deep rooting (associated with drought tolerance) and resistance to the leaf spot disease. This newly generated lablab assembly is a valuable resource that will aid in the ongoing efforts for its genetic improvement and will be useful for future comparative genomics studies of the legume species.

## 2 Materials and methods

### 2.1 DNA/RNA isolation

For genome sequencing, we collected young leaf samples from a 60-day-old *L. purpureus* plant (accession PK2022T020), flash frozen and stored in liquid nitrogen until the extraction. The high molecular weight DNA was isolated using the Qiagen Genomic-tip 20/G according to the manufacturer’s protocol (Qiagen, Hilden, Germany). Evaluation of DNA quality and quantity was performed using the Pippin Pulse Electrophoresis System (Sage Science, Beverly, USA) and the Qubit 4 Fluorometer (Thermo Fisher Scientific, Waltham, USA), respectively, prior to the library construction.

We also obtained RNA sequence data from leaf tissues, 1-week-old and 3-week-old pods to assist with the downstream annotation process. Tissues for transcriptome sequencing were collected from the same individual used for genome sequencing. Tissue samples were immediately frozen and stored in liquid nitrogen until extraction. Total RNA was isolated following the protocol reported in (Pootakham et al., 2023). Briefly, the CTAB buffer and 25:24:1 phenol:chloroform:isoamyl alcohol were used to extract RNA, which was subsequently precipitated overnight in ¼ volume of 8M LiCl. RNA pellets were washed with 70% ethanol, air-dried and resuspended in RNase-free water. The RNA integrity was evaluated using the Fragment Analyzer system (Agilent, Santa Clara, USA) prior to RNA sequencing library construction.

### 2.2 Genome and transcriptome sequencing

To generate the preliminary draft genome assembly, we constructed the stLFR sequencing library using a total of 10 ng of high molecular weight DNA following the MGIEasy stLFR Library Prep Kit’s instruction (MGI Tech, Shenzhen, China). For transcriptome sequencing, 200 ng of total RNA samples were used to construct the libraries using the MGIEasy RNA Library Prep Kit v3.0 according to the manufacturer’s protocol (MGI Tech, Shenzhen, China). Both stLFR and RNA libraries were sequenced on the DNBSEQ-G400 using the MGISEQ-2000RS Sequencing Flow Cell v3.0 (MGI Tech, Shenzhen, China).

### 2.3 Lablab genome assembly and Hi-C scaffolding

The preliminary draft genome was assembled from the 150-bp paired-end sequencing data using the single-tube long fragment read data analysis software stLFRdenovo v1.0.5 available from https://github.com/BGI-biotools/stLFRdenovo/releases/tag/v1.0.5. This preliminary assembly was further scaffolded into a chromosome-level assembly using the chromosome conformation capturing technique (Hi-C), which was conducted by Biomarker Technologies Corporation (Beijing, China). We assessed the sequence quality of the final assembly by aligning short-read DNA (from the stLFR library) and RNA sequencing data to the genome using BWA version 0.7.17 (Li and Durbin, 2009) for DNA sequence alignment and HISAT2 version 2.2.0 (Kim et al., 2019) for RNA sequence alignment. Furthermore, the completeness of the gene space was evaluated against the Embryophyta OrthoDB release 10 (Kriventseva et al., 2015) using the Benchmarking Universal Single-Copy Orthologs (BUSCO) version 5.4.4 (Manni et al., 2021). Short-read DNA sequences from our lablab accession PK2022T020 were aligned to the published genome (Njaci et al., 2023) using BWA version 0.7.17 (Li and Durbin, 2009), and GATK HaplotypeCaller version 4.1.4.1 (McKenna et al., 2010) with the Best Practices workflow was used to discover single nucleotide polymorphisms (SNPs) present between the two varieties (at the depth coverage between 20X and 200X). Subsequently, SnpEff version 5.2 was employed to annotate the variants and predict their functional effects (Cingolani et al., 2012).

### 2.4 Repeat element and gene annotation

We first generated a *de novo* repeat library using the RepeatModeler software version 2.0.3 (https://www.repeatmasker.org/RepeatModeler/) in order to identify transposable element (TE) families in the assembly (Flynn et al., 2020). This package consisted of three *de novo* repeat finding programs: RECON, RepeatScout and LtrHarvest/Ltr_retriever, which utilized complementary approaches to identify TE boundaries (Price et al., 2005; Bao and Eddy, 2002). After we obtained the repeat library, we aligned the repeat sequences to NCBI GenBank’s non-redundant protein database using BLASTX with the e-value cutoff of 10^-6^ to verify that the library did not contain sequences belonging to large families of protein-coding sequences.

To annotate protein-coding sequences, we employed the EVidenceModeler (EVM) software version 1.1.1 (Haas et al., 2008), which allowed a flexible combination of various evidence types into a single automated annotation system. We combined three evidence types to annotate the unmasked assembly: homology-based prediction, RNA-based prediction and *ab initio* prediction. For transcript-based prediction, we used evidence from the RNA-seq data obtained from leaf, tissues, 1-week-old and 3-week-old pods. Raw reads were first assembled into transcripts using Trinity version 2.9.1 (Haas et al., 2013) and clustered at a 95% identity with CD-HIT version 4.8.1. The longest ORF from each cluster was chosen as a representative to align with the genome assembly using PASA version 2.5.3 (Haas et al., 2003) and genomic mapping and alignment program (GMAP) version 2020-09-12 (Wu and Watanabe, 2005). Protein sequences from *Phaseolus vulgaris* (GCF_000499845.1), *Vigna angularis* (GCF_016808095.1), *Cajanus cajan* (GCF_000340665.2), *Medicago truncatula* (GCF_003473485.1), *Arabidopsis thaliana* (GCF_000001735.4) and *L. purpureus* (https://doi.org/10.5447/ipk/2022/26) available on the public databases were aligned to the genome assembly using the AAT (analysis and annotation tool) (Huang et al., 1997). *Ab initio* protein-coding gene predictions were obtained with Augustus version 3.2.1 trained with *P. vulgaris, V. angularis, C. cajan, M. truncatula, A. thaliana, L. purpureus* and PASA transcriptome alignment assembly using *L. purpureus* alignment files as inputs. Three types of evidence were integrated by EVM to generate consensus gene models using the following weights for each type: PASA – 5, GMAP – 1, AAT – 0.5, Augustus – 0.1.

### 2.5 Comparative genomics and phylogenetic analyses

We identified orthologous groups in *A. thaliana, Citrullus lanatus, Cucumis melo, Cucumis sativus, Glycine max, L. purpureus, P. vulgaris, Vigna unguiculata, Vigna reflexo-pilosa, Vigna hirtello, Vigna trinervia, Vigna radiata* and *Vigna mungo* using OrthoFinder version 2.4.0 (Emms and Kelly, 2019) and constructed a phylogenetic tree based on protein sequences from single-copy orthologous groups using RAxML-NG software version 1.0.2 (Stamatakis, 2006). Protein sequences from each single-copy orthologous group were aligned with MUSCLE version 3.8.1551 (Edgar, 2004), and alignment gaps were removed with trimAl version 1.4 rev15 (Capella-Gutiérrez et al., 2009). The alignment blocks were concatenated using the catsequences program (https://github.com/ChrisCreevey/catsequences), and the best-fit model of each block was selected using the ModelTest-NG software version 0.1.7 (Darriba et al., 2020). The outputs were subsequently used to compute a maximum likelihood phylogenetic tree. We estimated the species divergence time using the MCMCtree program in the software PAML 4 (Yang, 2007) using the relaxed-clock model with the known divergence time between *C. sativus* and *C. melo*, estimated to be at 8.4 to 11.8 million years ago (MYA) (Sebastian et al., 2010). The expansion and contraction analysis of the gene family was performed using CAFE [version 5.0 (De Bie et al., 2006)], which necessitates the presence of at least one gene within each family at the root of the phylogenetic tree. Gene families not meeting this criterion were excluded from the subsequent analysis.

### 2.6 Genome synteny analysis

We analyzed the collinearity within the *L. purpureus* genome and between *L. purpureus* – *Vigna angularis* (Adzuki bean) and *L. purpureus* – *V. unguiculata* (cowpea) genomes using MCscanX (Wang et al., 2012). We aligned *L. purpureus* amino acid sequences against themselves using BLASTP with an e-value cutoff of 10^-10^ in order to identify putative paralogues. Intragenic homologous regions were defined as sequences of at least ten genes with colinear runs of paralogues elsewhere in the genome with fewer than six intervening genes. Pairwise comparisons of input protein sequences among *L. purpureus, V. angularis* and *V. unguiculata* were performed using BLASTP with an e-value cutoff of 10^-10^ to identify putative orthologues. Clustering was carried out based on the Markov clustering algorithm (MCL) using the OrthoMCL software version 2.0.9 (Li et al., 2003). Syntenic regions between two genomes were identified with MCscanX using similar criteria applied for the intragenic homologous regions (at least ten colinear genes and no more than six intervening genes). Intragenic homologous regions in the *L. purpureus* genome and syntenic regions between *L. purpureus – V. angularis* and *L. purpureus – V. unguiculata* genomes were plotted with CIRCOS version 0.69.8 (Krzywinski et al., 2009).

## 3 Results

### 3.1 Genome assembly and evaluation

**Figure 1:**
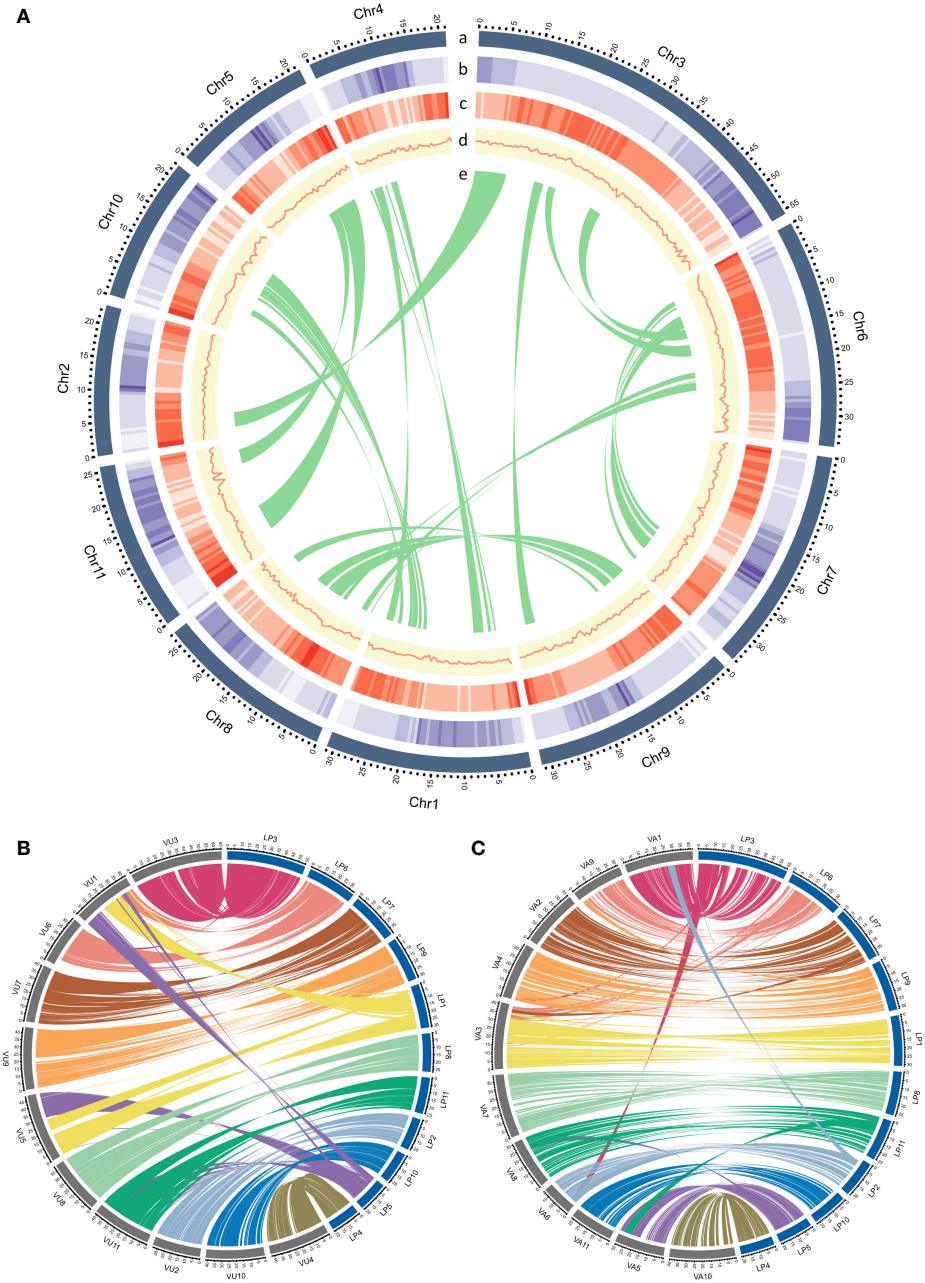
**(A)** Genomic landscape of *L. purpureus*. Concentric circles illustrate the following (from the outermost layer inwards): (a) a physical map of 11 chromosomes numbered according to size (Mb), (b) repeat density represented by the fraction of genomic regions covered by repetitive sequences in 250-kb windows, (c) gene density represented by the number of genes in 250-kb windows, (d) GC content represented by the percentage of G + C bases in 250-kb windows, (e) syntenic regions in the genome are shown by connected lines. **(B, C)** displayed synteny between *L. purpureus – V. unguiculata* and *L. purpureus – V. angularis*, respectively.

To achieve the chromosome-scale assembly of *L. purpureus* genome, we combined the linked-read stLFR technique and chromatin conformation capture (Hi-C) technology. Initially, we generated a preliminary assembly from 101.6 Gb of stLFR sequencing data. The stLFR technology enables sequencing of data from long DNA molecules by adding the same barcode sequence to sub-fragments of the original long DNA molecule (Wang et al., 2019). Our preliminary assembly had a total length of 367,397,371 bases, and the assembled scaffolds feature an N50 (L50) of 4,335,588 bases (Haas et al., 2008; **Table 1**). The subsequent application of the Hi-C method further scaffolded the draft assembly into a more contiguous, chromosome-level version. The final assembly comprised 366,384,401 bases with an N50 (L50) of 31,125,449 (Hardallo et al., 1980; **Table 1**). Notably, 89.4% of the final assembly (327.47 Mb) was successfully anchored into 11 pseudochromosomes (hereafter referred to as chromosomes; **Table 1**, **Figure 1**), mirroring the haploid chromosome number in lablab (2*n* = 2*x* =22). The chromosomes were numbered according to (Njaci et al., 2023). The assembly sizes reported in previous studies for *L. purpureus* were 395.5 Mb (Chang et al., 2019) and 426.2 Mb (cv. Highworth) (Njaci et al., 2023), slightly larger than our assembly for accession PK2022T020.

**Table 1:** *L. purpureus* genome assembly statistics.

	stLFR sequencing	stLFR sequencing + Hi-C
N50 contig/scaffold size (bases)	4,335,588	31,125,449
L50 contig/scaffold number	23	5
N75 contig/scaffold size (bases)	1,433,188	22,083,820
L75 contig/scaffold number	54	9
N90 contig/scaffold size (bases)	32,532	368,323
L90 contig/scaffold number	300	15
Assembly size (bases)	367,397,371	366,384,401
Number of scaffolds	8,338	7,670
Number of scaffolds ≥ 100 kb	163	22
Number of scaffolds ≥ 1 Mb	63	11
Number of scaffolds ≥ 10 Mb	8	11
Longest scaffold (bases)	13,153,325	55,630,846
% N	2.29	2.31
GC content (%)	30.44	30.44
BUSCO evaluation (% completeness)	98.2	98.4

The BUSCO assessment of the gene space completeness using 1,614 Embryophyta (plant-specific) single-copy orthologs revealed that the proportions of complete (C), complete and single-copy (S), complete and duplicated (D), fragmented (F) and missing (M) genes in our *L. purpureus* assembly were C:98.4% [S:96.0%,D:2.4%], F:0.3%, M:1.3%, respectively. We also evaluated the quality of our assembly by aligning whole genome sequence reads (stLFR sequence data) and RNA sequence data to the genome. The overall mapping rate of the whole genome sequence reads was 97.97% and that of the RNA-seq data was 97.26%, suggesting that our genome assembly is of high accuracy. Examination of synteny between lablab and two warm-season legume species revealed extensive conservation between *L. purpureus* and *V. unguiculata* and *L. purpureus* and *V. angularis* (**Figure 1**).

A comparison between our assembly (PK2022T020) and the previously published genome (Highworth) revealed a total of 191,290 SNP variants, reflecting a change rate of 1 in every 2,184 bases. The majority of the SNPs (56.59%) was present in intergenic regions (**Supplementary Figure S1**). Similar proportions of SNPs were identified in the upstream (15.99%) and downstream (15.84%) regions flanking the genes. Only 2.55% and 7.51% of the variants were detected in the exons and introns, respectively. Among the 7,225 SNP loci discovered in the exons, missense (4,049; 56.04%) and silent (3.086; 42.71%) mutations were the predominant classes while nonsense mutations represented a minor fraction (1.24%; **Supplementary Figure S1**). In addition to the single nucleotide variants observed, we identified chromosomal inversions in PK2022T020 compared to the Highworth reference genome as shown in the dot plot (**Supplementary Figure S2**). We found evidence of paired-end read alignments that extended across the junctions to support our Hi-C assembly. Over the past few decades, the comparative analyses of genetic linkage maps and genomic approaches have revealed that inversions are ubiquitous across plant and animal kingdoms (Wellenreuther and Bernatchez, 2018). Chromosomal inversions have been known to segregate in natural populations of various plant species including Arabidopsis (Zapata et al., 2016), sorghum (Deschamps et al., 2018), barley (Himmelbach et al., 2018) and honeysuckle (Yu et al., 2022). They are believed to play a crucial role in facilitating local adaptations by reducing recombination between favorable combinations of alleles (Thorstensen et al., 2022).

### 3.2 Genome annotation

Based on *de novo* prediction and homology-based repeat identification approaches, a total of 143.8 Mb of repetitive sequences were identified in the *L. purpureus* genome, accounting for 39.2% of the assembly (**Table 2**). The repeat content of our assembly was comparable to the figures previously reported [37.18% by (Chang et al., 2019) and 43.4% by (Njaci et al., 2023)]. DNA transposons and retrotransposons constituted the majority of known repeats, representing 6.2% and 33.2% of the total repeat contents, respectively. Intriguingly, more than half of the repetitive sequences (54.6%) in the *L. purpureus* genome were unclassified (**Table 2**).

**Table 2:** Repeat elements in the *L. purpureus* genome assembly.

Types of repeats	Bases (Mb)	% of the assembly	% of total repeats
**DNA transposons:**	8.93	2.44	6.21
**Retrotransposons:**			
LINE	1.39	0.38	0.96
SINE	0.0034	0.00	0.00
LTR: *Copia*	34.55	9.43	24.03
LTR: *Gypsy*	11.34	3.09	7.88
LTR: Others	0.44	0.12	0.30
**Simple sequence repeats:**	8.62	2.35	5.99
**Others:**	78.49	21.43	54.63
**Total**	143.77	39.24	

Employing *ab initio* based, homology-based and transcript-based methods, we predicted 28,511 gene models in *L. purpureus*, of which 26,441 were protein-coding genes. The mean length of predicted mRNAs were 3,317 bases, and the average number of exons per gene was 5.13 (**Supplementary Table S1**). Among the 26,441 protein-coding genes, 22,082 predicted genes were supported by the RNA-seq expression data (FPKM > 0.05). Functional annotation of predicted genes showed that 23,236 genes were assigned gene ontology (GO) terms (**Supplementary Table S2**). The most prevalent terms associated with biological process, cellular component and molecular function were regulation of DNA-templated transcription, membrane and ATP binding, respectively (**Supplementary Figure S3**). Additionally, 20,064, 11,524 and 5,161 genes were annotated with the Swissprot, EC and KEGG databases, respectively (**Supplementary Table S2**). Noncoding RNA prediction identified a total of 37,830 ncRNAs (2.87 Mb), comprising 317 rRNAs, 847 tRNAs, 4,132 miRNAs and 13,780 snRNAs (**Supplementary Table S3**).

### 3.3 Phylogenetic and comparative genomics analyses

**Figure 2:**
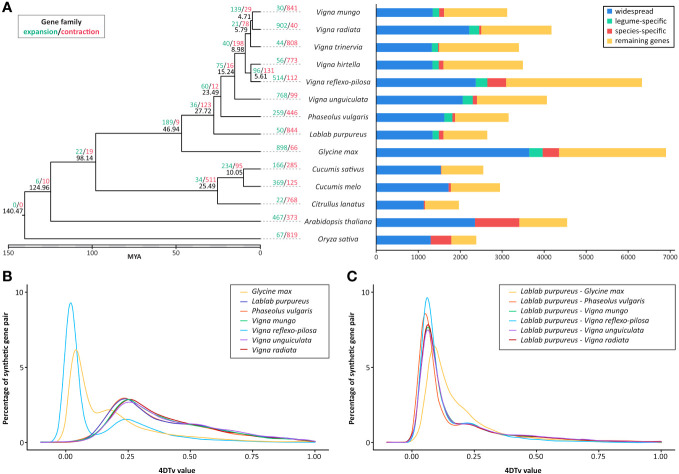
Comparative genomics of *L. purpureus*, related Fabaceae species and other plant species. **(A)** Maximum-likelihood phylogenetic tree of *L. purpureus* and other Fabaceae species using single-copy orthologous protein sequences. Numbers at each node (in black) represent the estimated divergence time in MYA. The number of expanded and contracted gene families in indicated in green and red, respectively. Bar charts show the number of proteins that were widespread (found in all species analyzed), legume-specific and species-specific. Distribution of 4DTv distances between orthologous genes **(B)** and paralogous genes **(C)** in *L. purpureus, G. max, P. vulgaris, V. mungo, V. reflexo-pilosa, V. unguiculata* and *V. radiata.*.

To determine the evolutionary relationship between *L. purpureus* and other plant species, a total of 517,182 proteins (out of 545,339 input proteins from 14 representative species; 94.84%) were clustered into 36,712 orthologous groups and used to generate a maximum-likelihood phylogenetic tree with *O. sativa* as an outgroup. The phylogenetic tree illustrated that *L. purpureus* diverged from the last common ancestor of the *Phaseolus/Vigna* species approximately 27.7 MYA (**Figure 2**).

Gene family expansion and contraction analysis across nine bean species and five other plant species identified 189 significantly expanded and 9 significantly contracted gene families in Fabaceae (out of the 36,712 gene families identified among species analyzed; **Figure 2**). *L. purpureus* exhibited 50 significantly expanded and 844 significantly contracted gene families. A large number of expanded gene families were associated with responses to biotic and abiotic stresses such as the leucine-rich repeat receptor-like protein kinase, disease resistance protein RGA2-like, G-type lectin S-receptor Ser/Thr kinase, salicylic acid binding protein and ethylene-responsive transcription factor (**Supplementary Table S4**). Among the significantly contracted gene families were those functioning in the signal transduction pathway, for instance, Ser/Thr kinases, proline-rich receptor-like PERK9 kinases, shaggy-related protein kinase, L-type lectin-domain containing receptor kinases and Ser/Thr phosphatase PP1 (**Supplementary Table S4**).

We employed the 4DTv approach, which measures the transversion rate at four-fold degenerate synonymous sites, to analyze the orthologous gene pairs in order to estimate the relative timing of evolutionary divergence between *L. purpureus* and closely related legume species (**Figure 2**). The result showed that the speciation between *G. max* and the last common ancestor of *L. purpureus* and *Phaseolus/Vigna* species occurred before the speciation events that separated *L. purpureus* from other legume species analyzed. The distribution of 4DTvs among paralogous gene pairs indicated no evidence of whole genome duplication events in all species except *V. reflexo-pilosa* and *G. max*.

## 4 Discussion

*L. purpureus* is an important tropical legume species widely cultivated as field and vegetable crops by small-farm holders throughout tropical regions in Asia. In our study, we successfully sequenced and assembled the reference genome of lablab accession PK2022T020, a landrace cultivar extensively grown for vegetable pod/seed consumption in Thailand. Utilizing the stLFR technique together with the chromatin contact mapping (Hi-C) technology, we achieved a chromosome-scale assembly of the lablab genome encompassing a total of 366 Mb. Our assembly contains 11 pseudochromosomes corresponding to lablab’s haploid chromosome number. Comparing our assembly to the previously reported genome of cultivar Highworth (426 Mb) (Njaci et al., 2023), our lablab assembly (accession PK2022T020) is slightly smaller; however, the completeness of the gene space measured by BUSCO are comparable between the two assemblies (98.4% for our assembly and 98.5% for Highworth), suggesting that both assemblies are of high quality. A comparison of SNP variants between the two genomes revealed several missense and nonsense mutations in the PK2022T020 accession that may potentially be associated with the phenotypic differences between these two varieties. LTRs were the predominant class of our lablab assembly, constituting nearly one third of the total repetitive sequences in the genome. Interestingly, the proportion of *Copia* LTRs exceeded that of the *Gypsy* LTRs, uncommon occurrences among lablab’s relative species (Pootakham et al., 2023; Kang et al., 2014; Pootakham et al., 2021; Schmutz et al., 2014). Examining expanded gene families in *L. purpureus*, our findings align with previous observations that indicated enrichment in genes associated with responses to biotic and abiotic stresses (Njaci et al., 2023). Obtaining lablab cultivars with superior tolerance to biotic and abiotic stresses has consistently been a primary breeding objective. We strongly believe that the availability of a chromosome-scale reference genome for PK2022T020 will play a pivotal role in advancing our understanding of lablab biology and greatly facilitating its molecular breeding programs that ultimately lead to the development of elite cultivars globally.

## Data availability statement

*L. purpureus* genome assembly and transcriptome data have been submitted to the DDBJ/EMBL/GenBank databases under Bioproject PRJNA1008422 and the following accession numbers: JAVGVT000000000 (genome assembly), SRR26115218 (RNA-seq; leaf), SRR26115216 (RNA-seq; 1-week-old pod), SRR26115215 (RNA-seq; 3-week-old pod), SRR26115217 (RNA-seq; flower).

## Author contributions

WP: Conceptualization, Formal analysis, Investigation, Supervision, Writing – original draft, Writing – review & editing. PS: Conceptualization, Methodology, Writing – original draft. WK: Formal analysis, Methodology, Visualization, Writing – review & editing. CN: Formal analysis, Methodology, Writing – review & editing. CS: Formal analysis, Methodology, Writing – review & editing. SU: Methodology, Writing – review & editing. TY: Methodology, Writing – review & editing. PP: Methodology, Writing – review & editing. ST: Conceptualization, Funding acquisition, Investigation, Supervision, Writing – review & editing.
