# Supplementary material for: A de novo chromosome-scale assembly of the Lablab purpureus genome
Source: Front Plant Sci. 2024 Mar 5;15:1347744. doi: 10.3389/fpls.2024.1347744 (PMC10948561; doi:10.3389/fpls.2024.1347744)
Supplement: Supplementary file 1 [file DataSheet_1.pdf]

Supplementary Figures

A *de novo* chromosome-scale assembly of the *Lablab purpureus* genome

Wirulda Pootakham[1], Prakit Somta[2], Wasitthee Kongkachana[1], Chaiwat Naktang[1], Chutima Sonthirod[1], Sonicha U-Thoomporn[1], Thippawan Yoocha[1], Poompat Phadphon[1], Sithichoke Tangphatsornruang[1,*].

[1]National Center for Genetic Engineering and Biotechnology (BIOTEC), National Science and Technology Development Agency (NSTDA), Pathum Thani, Thailand.
[2]Department of Agronomy, Faculty of Agriculture at Kamphaeng Saen, Kasetsart University, Nakhon Pathom, Thailand.

*Corresponding authors
Sithichoke Tangphatsornruang (sithichoke.tan@biotec.or.th)

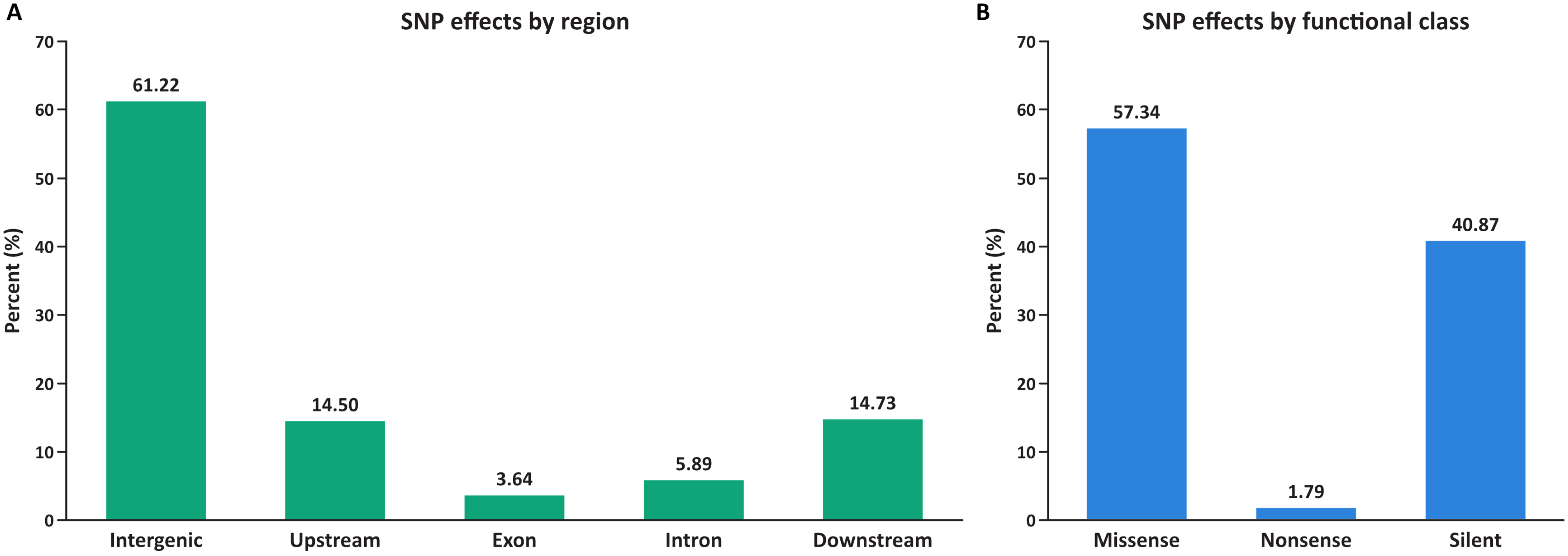

Supplementary Figure S1. Proportions of SNP effects by (A) region and (B) functional class.

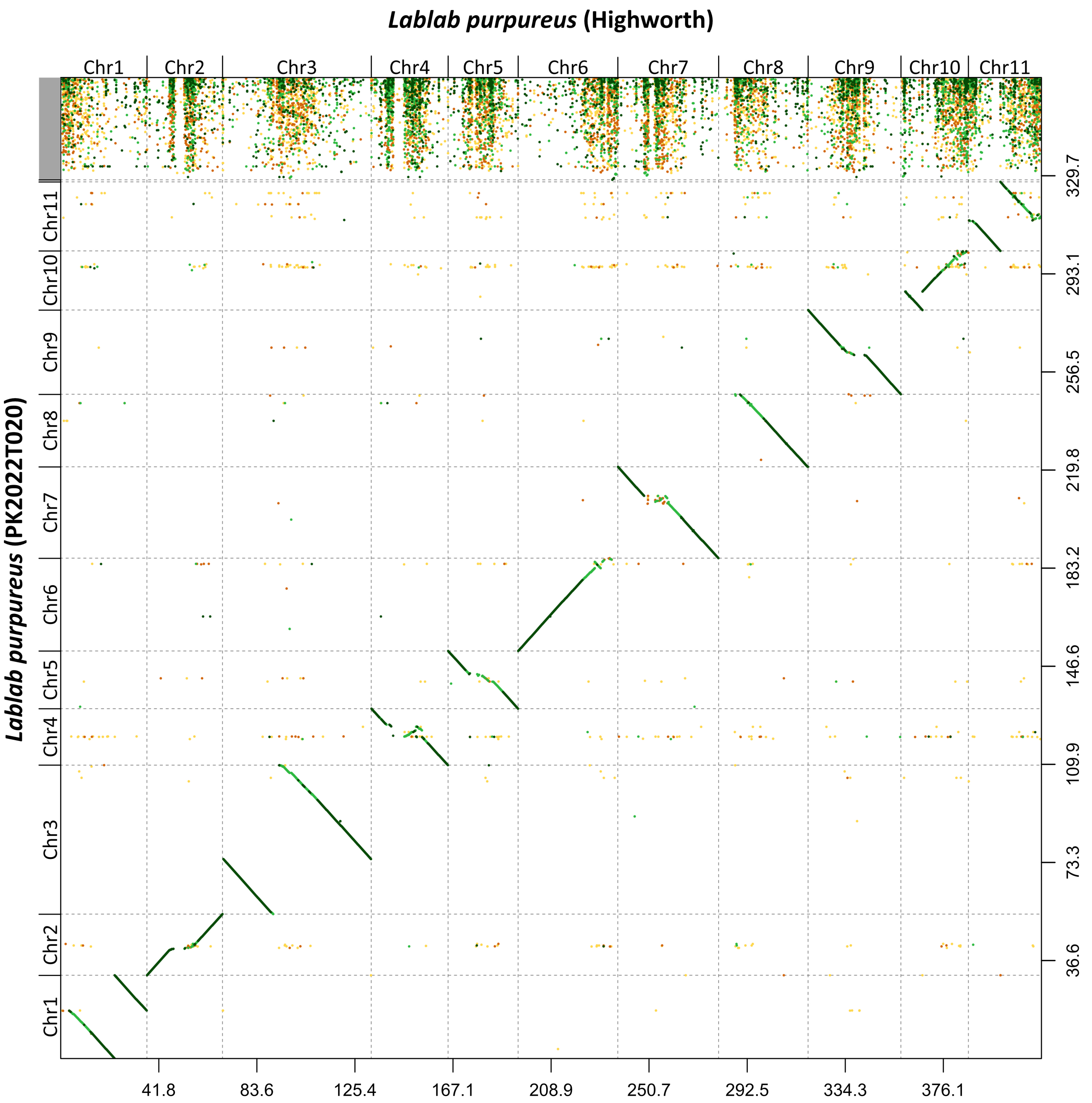

Supplementary Figure S2. A dot plot comparing lablab genome assemblies from two accessions: Highworth (Njaci et al, 2023) and PK2022T020 (this study).

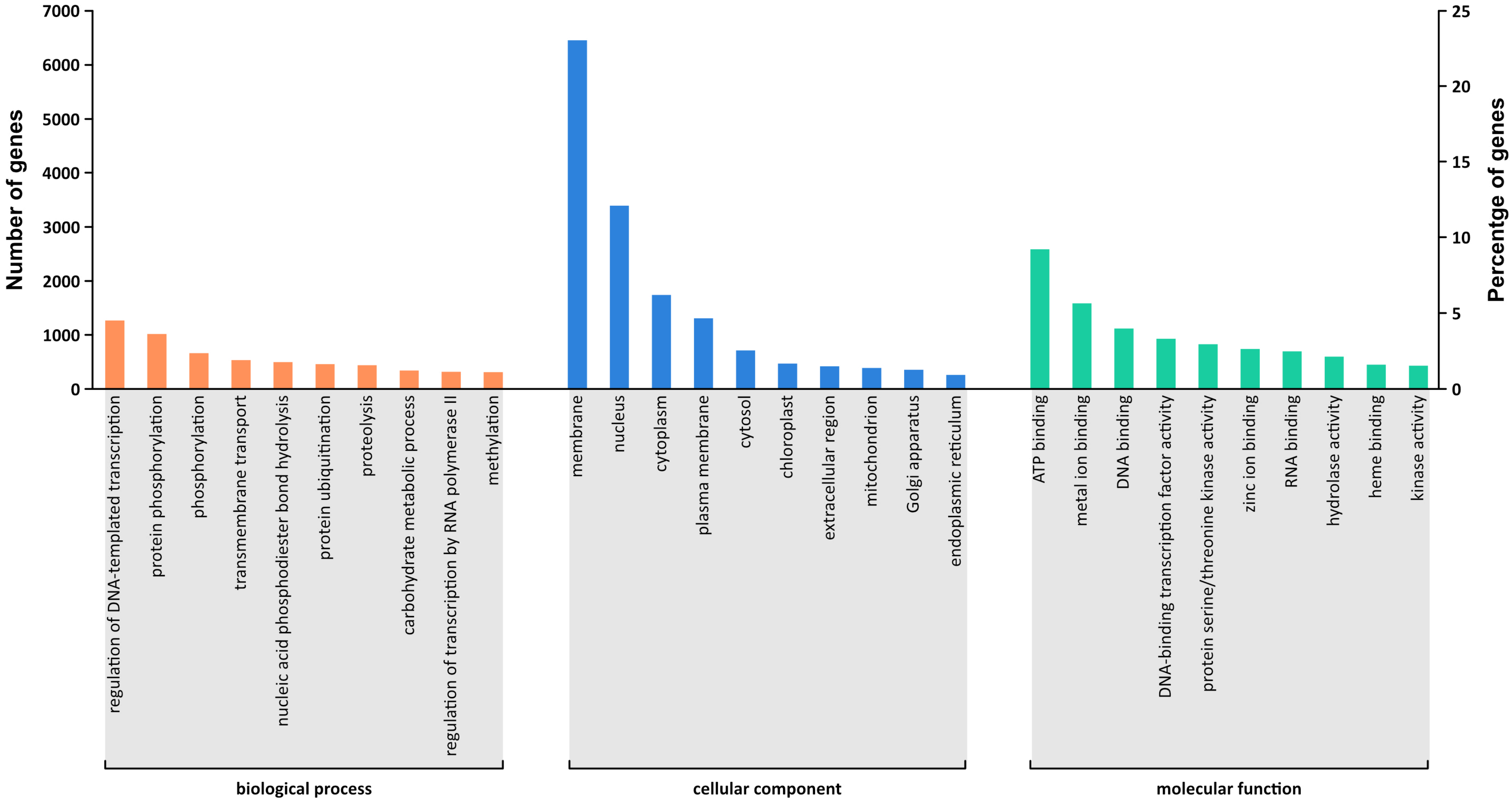

Supplementary Figure S3. Gene Ontology (GO) annotation of *L. purpureus* genes in the assembly.
